# Preoperative Plasma Fibrinogen and Serum Albumin Score Is an Independent Prognostic Factor for Resectable Stage II-III Gastric Cancer

**DOI:** 10.1155/2019/9060845

**Published:** 2019-10-29

**Authors:** Menghui Wu, Yuchen Pan, Zhifang Jia, Yueqi Wang, Na Yang, Jianfeng Mu, Tianyu Zhou, Yaohua Guo, Jing Jiang, Xueyuan Cao

**Affiliations:** ^1^Department of Gastric and Colorectal Surgery, The First Hospital of Jilin University, Changchun, China; ^2^Division of Clinical Research, The First Hospital of Jilin University, Changchun, China

## Abstract

**Background:**

Radical gastrectomy with D2 lymphadenectomy is recognized as the standard treatment for resectable advanced gastric cancer. Preoperative fibrinogen and albumin measurements may bring clinical benefits in terms of providing advanced notice of a poor prognosis or recurrence in patients undergoing radical resection. The aim of this study was to identify markers that are predictive of a poor prognosis prior to surgery.

**Methods:**

Eight hundred forty-two consecutive patients who underwent curative radical gastrectomy at our hospital between 2008 and 2012 were retrospectively reviewed. Based on plasma fibrinogen and serum albumin levels, preoperative fibrinogen and albumin scores (Fib-Alb scores) were investigated, and the prognostic significance was determined.

**Results:**

The patients were classified according to a Fib-Alb score of 0 (*n* = 376), 1 (*n* = 327), or 2 (*n* = 139). When the correlation between the response rate and the change in the Fib-Alb score was investigated, the response rate was significantly lower in patients with an increased Fib-Alb score than in the other patients. In the survival analysis, patients in the Fib-Alb high-score group exhibited significantly worse recurrence-free survival (RFS) (*P* = 0.030) than patients in the other groups. A multivariate analysis using clinical stage and the change in the Fib-Alb score as covariates revealed that a change in the Fib-Alb score (Fib-Alb score 1, HR: 1.31, 95% CI: 1.03-1.66, *P* = 0.028; Fib-Alb score 2, HR: 1.61, 95% CI: 1.20-2.17, *P* = 0.001) was a significant independent predictive factor for RFS.

**Conclusions:**

The prognosis of patients with high fibrinogen and low albumin levels is poor. The Fib-Alb score was shown to be an independent prognostic factor for postoperative recurrence in gastric cancer patients who underwent radical gastrectomy.

## 1. Introduction

According to the latest data, gastric cancer (GC) is the sixth most common malignancy worldwide, with an estimated 1.2 million incident cases and 834,000 deaths occurring in 2016 [1]. GC is the third-leading cause of cancer-associated morbidity and mortality in China [1]. Although GC patients typically undergo radical resection and postoperative adjuvant chemotherapy, the long-term survival rate of GC patients is far from satisfactory. Many studies have shown that tumour-related factors significantly affect the outcome and survival of GC patients, including the depth of invasion, lymph node metastasis, TNM stage, differentiation, vascular invasion, neural invasion, and distant metastasis [2–5]. There are many prognostic scoring systems based on blood tests that attempt to predict the recurrence of GC [6–9]. The factors affecting tumour prognosis mainly include hypercoagulability, nutritional status, and inflammation [8, 10–12]. Fibrinogen, a 340 kDa glycoprotein that is produced by hepatic cells, is converted to fibrin by activated thrombin and is an important product of the haemostatic system [13]. Recently, elevated preoperative fibrinogen levels were found to be correlated with a poor prognosis among GC patients [14, 15]. Serum albumin is produced in the liver and is an important factor that reflects nutritional status. Studies have shown that preoperative serum albumin levels are associated with the prognosis of several cancers, such as colorectal cancer, pancreatic cancer, ovarian cancer, and gastric cancer [16–22]. The level of serum albumin is also an important biomarker that is used to assess the nutritional status of GC patients [23]. It has been reported that preoperative fibrinogen and albumin levels could predict the prognosis of patients with esophageal cancer, hepatocellular carcinoma, breast cancer, non-small-cell lung cancer, and gallbladder cancer [24, 25]. To date, few studies have focused on the prognostic performance of the preoperative Fib-Alb score, and it is important to continue to explore the effects of Fib-Alb in large case studies. Thus, we performed a retrospective study to evaluate the correlations of the preoperative Fib-Alb score with clinicopathological characteristics and survival outcomes in GC patients. The present study also assessed the clinical applicability of the preoperative Fib-Alb score as a prognostic marker of GC.

## 2. Methods

### 2.1. Patients

Patients with histologically diagnosed GC who underwent gastrectomy with D2 lymphadenectomy at the Department of Gastric and Colorectal Surgery of the First Hospital of Jilin University between January 2008 and December 2012 were recruited for this study. Patients were excluded if they (1) underwent neoadjuvant therapy, (2) had previous malignancies or distant organ metastasis, (3) presented with positive margins, (4) were diagnosed with acute inflammatory disease, liver cirrhosis, chronic renal failure, autoimmune disease, or haematopoietic system disease, (5) died during the perioperative period or were lost to follow-up at 3 months after surgery, or (6) had stage IV GC. The TNM staging was determined based on the seventh edition of the *Cancer Staging Manual* of the Union for International Cancer Control/American Joint Committee on Cancer (UICC/AJCC). Informed consent was obtained from all the participants. This protocol was approved by the Ethics Committee of the First Hospital of Jilin University on February 19, 2013 (No. 2013-005). Neoadjuvant therapy may affect the levels of fibrinogen and albumin. Stage IV GC is considered difficult to resect via radical gastrectomy, which can affect patient survival. Thus, only patients who did not receive neoadjuvant therapy before gastrectomy and were not stage IV were enrolled in the analysis.

### 2.2. Fibrinogen and Albumin Measurements

Blood specimens were collected during the week before surgery. Plasma fibrinogen and serum albumin were measured immediately after blood sample collection. Plasma fibrinogen was assayed using Dade Thrombin Reagent (Siemens, Germany) and a CA7000 analyser (Sysmex Corporation), and the cumulative coefficient of variation (CV) was 7.3%. Serum albumin was measured using the bromocresol green (BCG) dye method and a biochemical analyser (7600-210, Hitachi, Japan), and the CV was 4.4% in the Clinical Laboratory of the First Hospital of Jilin University. Time-dependent receiver operating characteristic (ROC) analysis was performed to determine the optimal cut-off values of fibrinogen and albumin. Values above the optimal cut-off value were considered to be elevated, and those below the optimal cut-off value were considered to be decreased.

To reflect the effects of the two factors and the combined impact on GC prognosis, a scoring system of 2, 1, or 0 was chosen. Fib-Alb scoring was performed as follows: patients with elevated fibrinogen and decreased albumin levels were assigned a Fib-Alb score of 2; those with only one of these abnormalities were assigned a Fib-Alb score of 1; and those with neither of these abnormalities were assigned a Fib-Alb score of 0.

### 2.3. Follow-Up

Follow-up was scheduled at three, six, and twelve months after surgery, and then every year thereafter until the death of the patients or the end of the study. Abdominal computed tomography (CT) was used to determine the clinical staging. CT scanning was performed every 6 months in 2 years and every 12 months in 2 to 5 years after surgery. Information on the general status and on postoperative therapy was collected during each follow-up. Survival time was defined as the duration of the time from the date of surgery to the date of death by any cause or to the date of the last successful interview if the patient was still living or was lost to follow-up.

### 2.4. Statistical Analysis

Continuous variables are described using the median (range), and categorical variables are described as the frequency (percentage). Optimal cut-off values of fibrinogen and albumin were calculated by time-dependent ROC curves using R language, where the difference between a true positive (TP) and false positive (FP) was maximal for the prediction of 5-year survival. Survival curves were generated using the Kaplan-Meier survival method and compared by the log-rank test. Cox proportional hazards regression models were used to obtain the hazard ratios (HRs) and 95% CIs for evaluating the influence of possible factors on GC survival. Multivariate stepwise Cox regression analysis was also performed to select variables that independently predicted GC survival. Statistical analyses were performed with SPSS version 18.0 (SPSS Inc., Chicago, IL) and using R language (http://www.r-project.org). All *P* values were 2-tailed, and a *P* value < 0.05 was considered statistically significant.

## 3. Results

**Figure 1:**
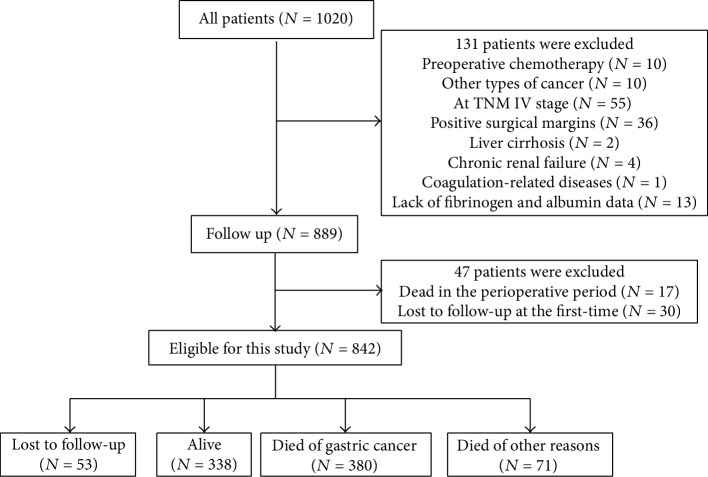
Flow of patients in the study.

A total of 842 patients were included in this study (Figure 1) and were followed up until September 2018. The median follow-up time was 83.9 months. During the follow-up period, 451 patients (53.6%) died, 338 patients (40.1%) remained alive, and 53 (6.3%) patients were lost to follow-up. Among the patients who died, 380 patients died as a result of GC, and 71 patients died as a result of other causes. The estimated 5-year survival rate was 55.5%.

The baseline characteristics of the patients are shown in Table 1. The mean age of the subjects was 60.5 ± 10.9 years, and 74.7% of the patients were male (629/842). In the current study, stage IV GC patients were excluded. Therefore, we divided the patients into two groups according to the TNM stage: stage I and stage II-III. Most of the patients (84.1%) were diagnosed at TNM stage II and stage III, and 15.9% of the patients were diagnosed at TNM stage I.

A total of 69.6% of the patients had poorly differentiated histopathological grading, and 30.4% had well or moderately differentiated grades. Nerve invasion was diagnosed by tumour cells invading any nerve sheath layer or tumours growing along the nerve. In the pathological section, a cluster of cancer cells found in lymphatic vessels and blood vessels and surrounded by endothelial cells was diagnosed as vascular invasion. Vascular and nerve invasion was observed in 73.3% and 55.2% of the patients, respectively. Tubular adenocarcinoma (85.4%) was the most common pathological type, followed by signet-ring cell carcinoma (8.9%) and others (5.7%). Only 30.6% of patients received 3 or more cycles of chemotherapy.

**Figure 2:**
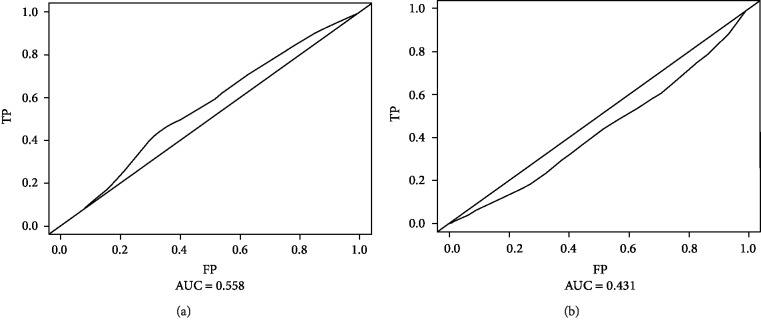
(a) Optimal cut-off values calculated by time-dependent receiver operating characteristic (ROC) for fibrinogen. (b) Optimal cut-off values calculated by time-dependent receiver operating characteristic (ROC) for albumin.

The median levels of plasma fibrinogen and serum albumin among the patients were 3.11 (1.14-10.91) g/L and 37.2 (4.6-50.5) g/L, respectively. The optimal cut-off values for fibrinogen and albumin to best predict prognosis were obtained with time-dependent ROC curve analysis: the optimal cut-off value for fibrinogen was 3.39 g/L (AUC: 0.558, sensitivity: 0.44, specificity: 0.67), and that for albumin was 34.9 g/L (AUC: 0.431, sensitivity: 0.61, specificity: 0.29) (Figures 2(a) and 2(b)). Nearly half (44.7%) of the patients had a Fib-Alb score of 0, followed by a Fib-Alb score of 1 (38.8%), with a Fib-Alb score of 2 being the least common (16.5%).

**Figure 3:**
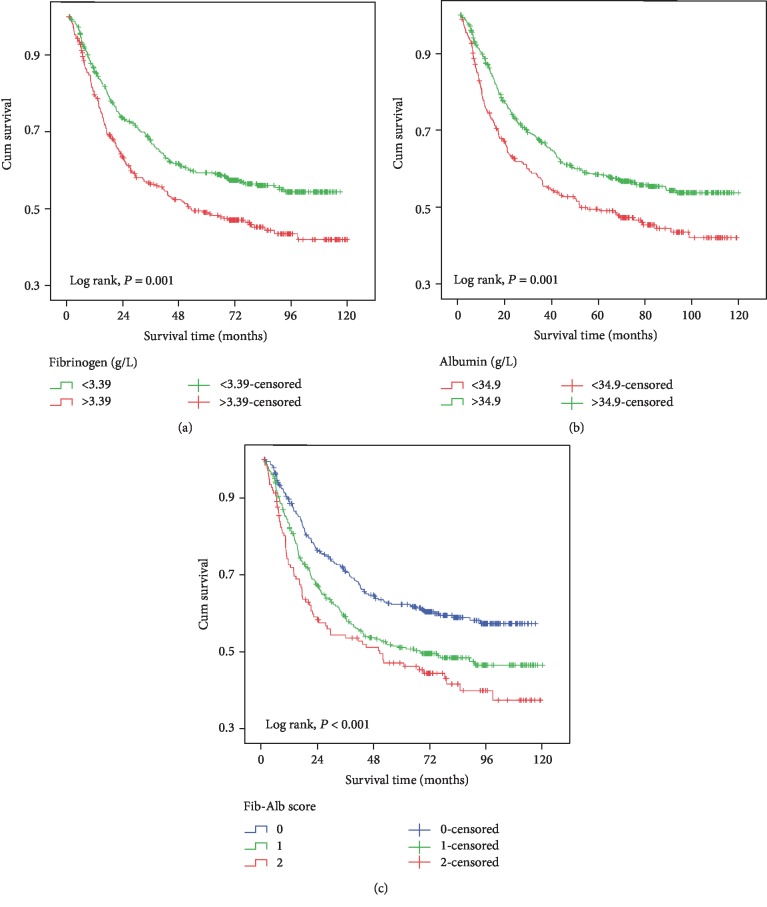
Kaplan-Meier curves of gastric cancer patient survival (log-rank test): (a) fibrinogen; (b) albumin; (c) Fib-Alb score.

The results of the univariate analysis are shown in Table 2 and in Figure 3. Patients with a higher fibrinogen level (>3.39 g/L) had a worse prognosis (HR: 1.41, 95% CI: 1.15-1.73, *P* = 0.001) (Figure 3(a)) than the other patients. The patients also tended to have a shorter survival if their albumin level was lower than 34.9 g/L (HR: 1.40, 95% CI: 1.14-1.13, *P* = 0.001) (Figure 3(b)) compared to that of the other patients. Compared with patients assigned a Fib-Alb score of 0, those who were assigned a score of 1 (HR: 1.42, 95% CI: 1.14-1.79, *P* = 0.002) or 2 (HR: 1.76, 95% CI: 1.34-2.32, *P* < 0.001) (Figure 3(c)) had a worse prognosis, which showed a good dose-response relationship (*P* for trend < 0.001).

The multivariate analysis results of the factors are shown in Table 3. The Cox regression analysis showed that after adjusting for age, TNM stage, histological grade, vascular invasion, and neural invasion, the levels of fibrinogen and albumin did not predict survival (*P* = 0.056, *P* = 0.088). The Fib-Alb score was shown to be an independent prognostic factor for patients with resectable GC (*P* for trend = 0.004); the HRs of a Fib-Alb score of 1 and a Fib-Alb score of 2 were 1.31 (95% CI: 1.03-1.66) and 1.61 (95% CI: 1.20-2.17), respectively. Moreover, older age, high TNM stage, positive vascular invasion, and positive neural invasion were also independent risk factors for overall survival.

**Figure 4:**
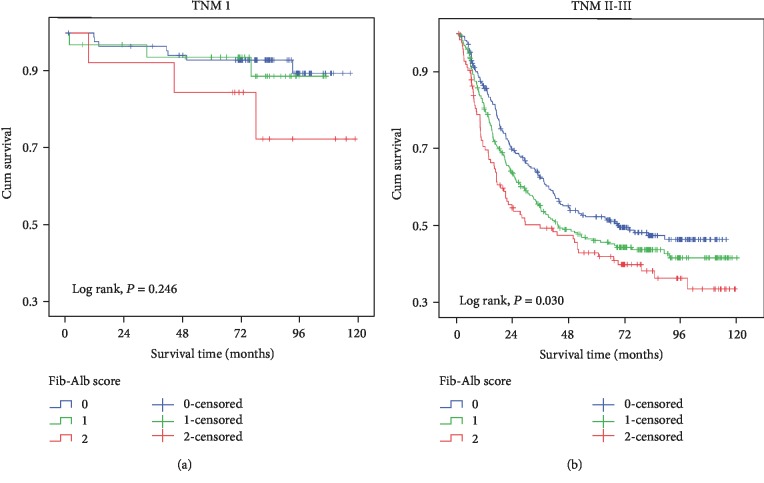
Survival curves of gastric cancer patients stratified by TNM stage. (a) Fib-Alb score in patients of TNM I stage. (b) Fib-Alb score in patients of TNM II-III stage.

The results of the stratification analysis are shown in Table 4. Among the patients with TNM stage I disease, when compared with patients with Fib-Alb scores of 0 and 1, those with Fib-Alb scores of 2 tended to have a worse prognosis (Figure 4(a), *P* = 0.246). However, in the Cox regression model, this difference was not statistically significant (*P* = 0.111). In patients with TNM stage II-III disease, the results showed a trend that was consistent with that of the whole patient cohort; a higher Fib-Alb score was independently associated with worse survival (Fib-Alb score 1, HR: 1.38, 95% CI: 1.01-1.61; Fib-Alb score 2, HR: 1.56, 95% CI: 1.17-2.07) (Figure 4(b)).

A correlation analysis between the “fibrinogen”/“albumin”/“Fib-Alb score” and vascular invasion, pathological type, and histological grading was performed, and the results are shown in Table 5. We found that the positive rate of vascular invasion (*P* = 0.034) was higher in the patients with Fib-Alb scores of 2 than in those with Fib-Alb scores of 0 and 1, and this difference was statistically significant.

## 4. Discussion

The univariate analysis indicated that older age (>60 years old), TNM stage II-III disease, moderate or high histological grade, positive vascular invasion, positive neural invasion, high fibrinogen levels (>3.39 g/L), low albumin levels (<34.9 g/L), and a Fib-Alb score of 1 and 2 were associated with a worse prognosis in GC patients compared to a score of 0. Moreover, the multivariate analysis showed that the Fib-Alb score but not the fibrinogen and albumin level was a powerful prognostic indicator for overall survival in TNM stage II-III patients.

In this study, we used high fibrinogen (>3.39 g/L) or low albumin (<34.9 g/L) levels as prognostic indicators for GC patient survival. However, the combination of plasma fibrinogen and serum albumin levels was identified as a better predictor of prognosis (Fib-Alb score = 1: HR, 1.31; 95% CI, 1.03-1.66; *P* = 0.028; Fib-Alb score = 2: HR, 1.61; 95% CI, 1.20-2.17; *P* = 0.001). In our study, a higher Fib-Alb score was found to be associated with a number of clinicopathological characteristics of GC patients, such as older age, vascular invasion, neural invasion, and TNM stage, which were independent risk factors for OS in GC, indicating that a higher Fib-Alb score might be associated with GC aggressiveness and progression. Studies have also demonstrated that high fibrinogen and low albumin levels could predict the prognosis of patients with several types of cancers [24–28].

The Fib-Alb score has recently been used to evaluate the prognosis of various tumours and is considered to be a reflection of systemic inflammation and nutritional status [25–28]. However, the molecular mechanisms underlying the preoperative fibrinogen and albumin levels remain undefined. Fibrinogen, as one of the acute-phase proteins that is mainly produced by the liver, is greatly enhanced in response to infection or other inflammatory disorders. It can also be produced by malignant tumour cells and can participate in the formation of the extracellular matrix (ECM) [29–31]. Fibrinogen can promote the adhesion, proliferation, and migration of tumour cells by binding with vascular endothelial growth factor (VEGF) and fibroblast growth factor-2 (FGF-2) [30–32]. It is a dimeric molecule with multiple integrin and nonintegrin binding motifs that can be used as a molecular bridge for the adhesion between tumour cells, platelets, and endothelial cells [33, 34]. Moreover, platelet-fibrin(ogen) microthrombi can facilitate tumour cell metastasis by impeding natural killer cell-mediated apoptosis [35, 36]. Palumbo et al. reported that fibrinogen played an important role in the spontaneous metastasis of tumours in fibrinogen-deficient mice [37]. This may be the reason why a high fibrinogen level is indicative of a poor prognosis.

Albumin, as an independent prognostic indicator for malignancies, may not only reflect poor nutritional status but also participate in systemic inflammation. It has been proven that both inflammation and malnutrition can suppress albumin synthesis [38]. Cancer-associated malnutrition may lead to the impairment of immune function, decrease the effectiveness of treatment, and increase morbidity and mortality [39]. As part of the systemic inflammatory response to a tumour or from the tumour itself, inflammatory mediators are released, including interleukin-1 (IL-1), IL-6, tumour necrosis factor- (TNF-) *α*, and acute-phase reactants [40]. Thus, albumin could affect tumour prognosis. The current study focused on the albumin levels, which could partly reflect nutritional status. There are some other indicators that also reflect nutritional status, such as weight loss, thin skin-fold thickness, and lower haemoglobin (Hb) level, predicting poor survival outcomes in GC patients [41–43]. In our study, low Hb was associated with high fibrinogen and low albumin levels (Table 6). However, low Hb levels were not associated with prognosis in GC patients. Meanwhile, there is no relevant information regarding weight loss, skin-fold thickness, and Hb levels related to prognosis at present, which is a limitation in this paper.

Nevertheless, in the TNM stage I GC, the Fib-Alb score did not show a significant association with prognosis. This finding indicated that there was no significant change in systemic inflammation, tumour metastasis, or nutritional status in early-stage GC patients. From this perspective, the preoperative Fib-Alb score could also reflect the progression of GC. We calculated the data according to stage (I, II, and III); however, the results for the Fib-Alb score were negative in the multivariate analysis. Positive results were only found for TNM stage II and III patients. These divergent results may be due to the weak influence of Fib-Alb; the impacts on the prognosis are not found when the number of analysed cases is low. When TNM stage II and III patients were combined and the number of cases increased, their correlation with prognosis was shown to be significant. Thus, we combined stage II and stage III in the analyses.

Histological grading may reflect the aggressiveness of the tumour and thus indirectly reflect the tendency of cancer cells to metastasize due to events that are influenced by factors such as fibrinogen. In the current study, patients had a higher in TNM stage II-III (84.1%) and poorly differentiated histological grading (69.6%). The patients diagnosed with poorly differentiated histological types showed a poor 5-year survival rate compared to those with well or moderately differentiated histological types (53.3% vs. 60%, *P* = 0.04) in the univariate analysis (Table 2). However, further multivariate analysis showed that prognosis was not significantly associated with histological grading.

In our study, 258 patients (258/842, 30.6%) received 3 or more cycles of chemotherapy postoperatively. Although the 5-year survival rate showed an increasing trend, a significant difference was not found between the chemotherapy groups and nonchemotherapy groups in the univariate analysis (57.9% vs.54.4%, *P* = 0.192). This result may be due to inconsistencies in postoperative chemotherapy regimens and in the number of cycles. Another reason is that the number of cases is small; the survival results may change as the number of cases is increased. Zhang et al. reported that patients with TNM stage II-III disease and high preoperative fibrinogen/prealbumin ratio (FPR) values will benefit from neoadjuvant therapy [44]. They found that the FPR could precisely distinguish stage III patients who could benefit from adjuvant chemotherapy. However, similar results were not observed in our study. The inconsistency was most likely due to the following: First, 69.2% (249/360) of patients received chemotherapy in Zhang's study, which was significantly higher than the percentage in our study (30.2%, 258/842). Second, Zhang et al. retrospectively analysed 3-year OS, whereas we calculated the data for 5-year OS. Finally, they studied 360 patients with GC limited to TNM stage II and III disease, whereas we studied 842 patients with stage I to stage III disease. These factors may underlie the differences observed between the two studies.

In our study, due to limited data availability, we did not discuss the effects of perioperative blood transfusions on prognosis. Previous studies have found that blood transfusions in the perioperative period affect the survival of GC patients. Squires et al. demonstrated that perioperative blood transfusion could reduce the recurrence-free and overall survival rates of GC patients [45]. Kanda et al. also found that perioperative blood transfusions were associated with a poor prognosis in patients at surgical stage II/III [46]. However, Cui et al. found that perioperative blood transfusions did not increase the risk of a poor prognosis in GC patients [47]. Grasso et al. also reported that perioperative blood transfusions should be avoided because immunomodulatory effects may worsen the prognosis of these patients [48]. The effects of perioperative blood transfusion on GC prognosis should be studied in the future.

In conclusion, the preoperative Fib-Alb score was a powerful and significant independent prognostic indicator of advanced GC, especially in patients with stage II/III disease. However, the detailed mechanisms underlying how the Fib-Alb score affects GC should be examined in the future. Moreover, the prognostic value of the Fib-Alb score in advanced GC patients needs to be determined in a larger-scale prospective study.

## Figures and Tables

**Table 1:** Characteristics of the patients included in the study (*N* = 842).

Characteristics	*N* (%)
Age (years)^#^	60.5 ± 10.9
Gender	
Male	629 (74.7)
Female	213 (25.3)
Depth of invasion	
T1	94 (11.1)
T2	104 (12.4)
T3	524 (62.2)
T4	120 (14.3)
Lymph node metastasis	
N0	234 (27.8)
N1	219 (26.0)
N2	180 (21.4)
N3	209 (24.8)
TNM stage	
I	134 (15.9)
II	318 (37.8)
III	390 (46.3)
Histological grading	
Poorly differentiated	586 (69.6)
Well or moderately differentiated	256 (30.4)
Vascular invasion	
Positive	617 (73.3)
Negative	225 (26.7)
Neural invasion	
Positive	465 (55.2)
Negative	377 (44.8)
Pathological type	
Tubular adenocarcinoma	719 (85.4)
Signet-ring cell carcinoma	75 (8.9)
Other types	48 (5.7)
Chemotherapy	
No	584 (69.4)
Yes	258 (30.6)
Fibrinogen (g/L)^∗^	3.11 (1.14-10.91)
Albumin (g/L)^∗^	37.2 (4.6-50.5)
Fib-Alb score	
0	376 (44.7)
1	327 (38.8)
2	139 (16.5)

^#^Mean ± sd; ^∗^median (range).

**Table 2:** Univariate analysis of the factors associated with the prognosis of gastric cancer.

Variable	Classification	*N*	Death (%)	5-year survival rate	HR (95% CI)	*P*
Age (years)	≤60	434	177 (40.8)	59.6	1.00	
	>60	408	203 (49.8)	51.0	1.32 (1.08-1.61)	0.008

Gender	Male	629	288 (45.8)	55.0	1.00	
	Female	213	92 (43.2)	56.8	0.94 (0.74-1.18)	0.577

TNM stage	I	134	13 (9.7)	92.3	1.00	
	II	318	109 (34.3)	68.1	4.33 (2.44-7.69)	<0.001
	III	390	258 (66.2)	30.9	12.66 (7.24-22.14)	<0.001

Histological grading	Poorly differentiated	586	274 (46.8)	53.3	1.00	
	Well or moderately differentiated	256	106 (41.4)	60.4	0.79 (0.63-0.99)	0.040

Vascular invasion	Negative	225	45 (20.0)	81.9	1.00	
	Positive	617	335 (54.3)	45.5	3.69 (2.69-5.04)	<0.001

Neural invasion	Negative	377	115 (30.5)	71.2	1.00	
	Positive	465	265 (57.0)	42.2	2.49 (2.01-3.11)	<0.001

Pathological type	Tubular adenocarcinoma	719	325 (45.2)	55.9	1.00	
	Signet-ring cell carcinoma	75	31 (41.3)	56.7	0.92 (0.64-1.33)	0.646
	Other types	48	24 (50.0)	47.8	1.19 (0.79-1.81)	0.411

Chemotherapy	Yes	258	111 (43.0)	57.9	1.00	
	No	584	269 (46.1)	54.4	1.16 (0.93-1.45)	0.192

Fibrinogen	≤3.39	521	215 (41.3)	59.4	1.00	
	>3.39	321	165 (51.4)	49.0	1.41 (1.15-1.73)	0.001

Albumin	>34.9	558	232 (41.6)	58.5	1.00	
	≤34.9	284	148 (52.1)	49.5	1.40 (1.14-1.13)	0.001

Fib-Alb score^∗^	0	376	144 (38.3)	62.4	1.00	
	1	327	159 (48.6)	51.0	1.42 (1.14-1.79)	0.002
	2	139	77 (55.4)	47.1	1.76 (1.34-2.32)	<0.001

**Table 3:** Multivariate analysis of the factors associated with gastric cancer prognosis.

Variable	HR (95% CI)	*P*
Age (years)		
≤60	1.00	
>60	1.25 (1.01-1.55)	0.039
TNM stage		
I	1.00	
II-III	4.02 (2.26-7.16)	<0.001
Histological grading		
Poorly differentiated	1.00	
Well or moderately differentiated	1.01 (0.80-1.28)	0.907
Vascular invasion		
Negative	1.00	
Positive	2.22 (1.60-3.07)	<0.001
Neural invasion		
Negative	1.00	
Positive	1.71 (1.36-2.16)	<0.001
Fibrinogen		
≤3.39	1.00	
>3.39	1.22 (0.99-1.51)	0.056
Albumin		
>34.9	1.00	
≤34.9	1.21 (0.97-1.50)	0.088
Fib-Alb score^∗^		
0	1.00	
1	1.31 (1.03-1.66)	0.028
2	1.61 (1.20-2.17)	0.001

^∗^Adjusted age, TNM stage, histological grading, vascular invasion, and neural invasion.

**Table 4:** Multivariate analysis of the factors associated with gastric cancer prognosis in different TNM stages.

	Variable	*N*	HR (95% CI)	*P*
TNM stage I	Fib-Alb score			
	0	88	1.00	
	1	33	1.25 (0.32-4.82)	0.751
	2	13	3.00 (0.78-11.61)	0.111

TNM stage II-III	Fib-Alb score			
	0	288	1.00	
	1	294	1.38 (1.01-1.61)	0.038
	2	126	1.56 (1.17-2.07)	0.002

^∗^Adjusted age, histological grading, vascular invasion, and neural invasion.

**Table 5:** Correlation analysis between “fibrinogen”/“albumin”/“Fib-Alb score” and clinicopathological characteristics in GC cancer.

		Fibrinogen	*P*	Albumin	*P*	Fib-Alb score			*P*
						0	1	2	
Vascular invasion	Negative	3.31 ± 1.12	0.795	37.44 ± 5.55	0.181	117 (31.1)	77 (23.5)	31 (22.3)	0.034
	Positive	3.29 ± 0.89		36.89 ± 5.08		259 (68.9)	260 (76.5)	108 (77.7)	

Pathological type	Tubular adenocarcinoma	3.30 ± 0.93	0.505	36.95 ± 5.31	0.173	316 (84.0)	278 (85.0)	125 (89.9)	0.139
	Signet-ring cell carcinoma	3.18 ± 0.97		38.10 ± 3.61		42 (11.2)	26 (8.0)	7 (5.0)	
	Other types	3.38 ± 1.28		36.73 ± 5.69		18 (4.8)	23 (7.0)	7 (5.0)	

Histological grading	Poorly differentiated	3.26 ± 0.92	0.177	37.03 ± 5.26	0.967	274 (72.9)	217 (66.4)	95 (68.3)	0.163
	Well or moderately differentiated	3.36 ± 1.04		37.05 ± 3.12		102 (27.1)	110 (36.6)	44 (31.7)	

**Table 6:** Hb associated with fibrinogen and albumin levels.

		Fib (g/L)		*χ* ^2^	*P*	Alb (g/L)		*χ* ^2^	*P*
		≤3.39	>3.39			≤34.9	>34.9		
Hb (g/L)	30-60	0 (0)	1 (100)	12.08	0.004	0 (0)	1 (100)	142.18	<0.001
	60-90	37 (46.3)	43 (53.7)			57 (71.3)	23 (28.7)		
	90-120	132 (62.6)	79 (37.4)			112 (53.1)	99 (46.9)		
	>120	341 (65.3)	181 (34.7)			95 (18.2)	427 (81.8)		

## Data Availability

The analysed datasets in this study can be obtained from the corresponding authors upon reasonable request.

## References

[1] Global Burden of Disease Cancer Collaboration, Akinyemiju TF F. C., Al Lami F. H. (2018). Global, regional, and national cancer incidence, mortality, years of life lost, years lived with disability, and disability-adjusted life-years for 29 cancer groups, 1990 to 2016: a systematic analysis for the Global Burden of Disease Study. *JAMA Oncology*.

[2] Graham Martinez C., Knijn N., Verheij M., Nagtegaal I. D., van der Post R. S. (2019). Tumour deposits are a significant prognostic factor in gastric cancer – a systematic review and meta‐analysis. *Histopathology*.

[3] Li X., Liu Y., Cao B. (2015). Metastatic lymph node ratio and prognosis of gastric cancer at different pT stages. *Hepato-Gastroenterology*.

[4] Chiaravalli A. M., Klersy C., Vanoli A., Ferretti A., Capella C., Solcia E. (2012). Histotype-based prognostic classification of gastric cancer. *World Journal of Gastroenterology*.

[5] Nishibeppu K., Komatsu S., Ichikawa D. (2018). Venous invasion as a risk factor for recurrence after gastrectomy followed by chemotherapy for stage III gastric cancer. *BMC Cancer*.

[6] Takeno S., Hashimoto T., Shibata R. (2014). The high-sensitivity modified Glasgow prognostic score is superior to the modified Glasgow prognostic score as a prognostic predictor in patients with resectable gastric cancer. *Oncology*.

[7] Sun J., Wang D., Mei Y. (2017). Value of the prognostic nutritional index in advanced gastric cancer treated with preoperative chemotherapy. *The Journal of Surgical Research*.

[8] Sun K.-Y., Xu J.-B., Chen S.-L. (2015). Novel immunological and nutritional-based prognostic index for gastric cancer. *World Journal of Gastroenterology*.

[9] Hirahara N., Tajima Y., Fujii Y. (2019). Comprehensive analysis of red blood cell distribution width as a preoperative prognostic predictor in gastric cancer. *Anticancer Research*.

[10] Nozoe T., Ninomiya M., Maeda T., Matsukuma A., Nakashima H., Ezaki T. (2010). Prognostic nutritional index: a tool to predict the biological aggressiveness of gastric carcinoma. *Surgery Today*.

[11] Falanga A., Santoro A., Labianca R. (2016). Hypercoagulation screening as an innovative tool for risk assessment, early diagnosis and prognosis in cancer: the HYPERCAN study. *Thrombosis Research*.

[12] Repetto O., De Re V. (2017). Coagulation and fibrinolysis in gastric cancer. *Annals of the New York Academy of Sciences*.

[13] Yu X., Hu F., Yao Q., Li C., Zhang H., Xue Y. (2016). Serum fibrinogen levels are positively correlated with advanced tumor stage and poor survival in patients with gastric cancer undergoing gastrectomy: a large cohort retrospective study. *BMC Cancer*.

[14] Yu W., Wang Y., Shen B. (2016). An elevated preoperative plasma fibrinogen level is associated with poor overall survival in Chinese gastric cancer patients. *Cancer Epidemiology*.

[15] Ding P., Zheng C., Cao G. (2019). Combination of preoperative plasma fibrinogen and AJCC staging improves the accuracy of survival prediction for patients with stage I‐II gastric cancer after curative gastrectomy. *Cancer Medicine*.

[16] Heys S. D., Walker L. G., Deehan D. J., Eremin O. E. (1998). Serum albumin: a prognostic indicator in patients with colorectal cancer. *Journal of the Royal College of Surgeons of Edinburgh*.

[17] Boonpipattanapong T., Chewatanakornkul S. (2006). Preoperative carcinoembryonic antigen and albumin in predicting survival in patients with colon and rectal carcinomas. *Journal of Clinical Gastroenterology*.

[18] Sun L.-C., Chu K.-S., Cheng S.-C. (2009). Preoperative serum carcinoembryonic antigen, albumin and age are supplementary to UICC staging systems in predicting survival for colorectal cancer patients undergoing surgical treatment. *BMC Cancer*.

[19] Lai C.-C., You J.-F., Yeh C.-Y. (2011). Low preoperative serum albumin in colon cancer: a risk factor for poor outcome. *International journal of colorectal disease.*.

[20] Liu J., Chen S., Geng Q. (2017). Prognostic value of pretreatment albumin–globulin ratio in predicting long-term mortality in gastric cancer patients who underwent D2 resection. *OncoTargets and Therapy*.

[21] Lien Y. C., Hsieh C. C., Wu Y. C. (2004). Preoperative serum albumin level is a prognostic indicator for adenocarcinoma of the gastric cardia. *Journal of Gastrointestinal Surgery*.

[22] Saito H., Kono Y., Murakami Y. (2018). Postoperative serum albumin is a potential prognostic factor for older patients with gastric cancer. *Yonago Acta Medica*.

[23] Ouyang X., Dang Y., Zhang F., Huang Q. (2018). Low serum albumin correlates with poor survival in gastric cancer patients. *Clinical Laboratory*.

[24] Matsuda S., Takeuchi H., Kawakubo H. (2017). Prognostic impact of change in the fibrinogen and albumin score during preoperative treatment in esophageal cancer patients. *World Journal of Surgery*.

[25] Xu W.-Y., Zhang H.-H., Xiong J.-P. (2018). Prognostic significance of the fibrinogen-to-albumin ratio in gallbladder cancer patients. *World Journal of Gastroenterology*.

[26] Wang Y., Chen W., Hu C. (2017). Albumin and fibrinogen combined prognostic grade predicts prognosis of patients with prostate cancer. *Journal of Cancer*.

[27] Hwang K.-T., Chung J. K., Roh E. Y. (2017). Prognostic influence of preoperative fibrinogen to albumin ratio for breast cancer. *Journal of Breast Cancer*.

[28] Chen P., Wang C., Cheng B. (2017). Plasma fibrinogen and serum albumin levels (FA score) act as a promising prognostic indicator in non-small cell lung cancer. *OncoTargets and Therapy*.

[29] Simpson-Haidaris P. J., Rybarczyk B. (2001). Tumors and fibrinogen. The role of fibrinogen as an extracellular matrix protein. *Annals of the New York Academy of Sciences*.

[30] Sahni A., Khorana A. A., Baggs R. B., Peng H., Francis C. W. (2006). FGF-2 binding to fibrin(ogen) is required for augmented angiogenesis. *Blood*.

[31] Sahni A., Simpson-Haidaris P. J., Sahni S. K., Vaday G. G., Francis C. W. (2008). Fibrinogen synthesized by cancer cells augments the proliferative effect of fibroblast growth factor-2 (FGF-2). *Journal of Thrombosis and Haemostasis*.

[32] Sahni A., Francis C. W. (2000). Vascular endothelial growth factor binds to fibrinogen and fibrin and stimulates endothelial cell proliferation. *Blood*.

[33] Steinbrecher K. A., Horowitz N. A., Blevins E. A. (2010). Colitis-associated cancer is dependent on the interplay between the hemostatic and inflammatory systems and supported by integrin *α*_M_*β*_2_ engagement of fibrinogen. *Cancer Research*.

[34] Yano J. H., Hatano K., Tsuno N. (2001). Clustered cancer cells show a distinct adhesion behavior from single cell form under physiological shear conditions. *Journal of experimental & clinical cancer research: CR*.

[35] Palumbo J. S., Talmage K. E., Massari J. V. (2005). Platelets and fibrin(ogen) increase metastatic potential by impeding natural killer cell-mediated elimination of tumor cells. *Blood*.

[36] Zheng S., Shen J., Jiao Y. (2009). Platelets and fibrinogen facilitate each other in protecting tumor cells from natural killer cytotoxicity. *Cancer Science*.

[37] Palumbo J. S., Potter J. M., Kaplan L. S., Talmage K., Jackson D. G., Degen J. L. (2002). Spontaneous hematogenous and lymphatic metastasis, but not primary tumor growth or angiogenesis, is diminished in fibrinogen-deficient mice. *Cancer Research*.

[38] Yeun J. Y., Kaysen G. A. (1998). Factors influencing serum albumin in dialysis patients. *American Journal of Kidney Diseases*.

[39] Van Cutsem E., Arends J. (2005). The causes and consequences of cancer-associated malnutrition. *European Journal of Oncology Nursing*.

[40] Mantovani A., Allavena P., Sica A., Balkwill F. (2008). Cancer-related inflammation. *Nature*.

[41] Park Y. S., Park D. J., Lee Y. (2018). Prognostic roles of perioperative body mass index and weight loss in the long-term survival of gastric cancer patients. *Cancer Epidemiology Biomarkers & Prevention*.

[42] Guo Z. Q., Yu J. M., Li W. (2019). Survey and analysis of the nutritional status in hospitalized patients with malignant gastric tumors and its influence on the quality of life. *Supportive Care in Cancer*.

[43] Zhang S., Lu M., Li Y., Li J., Shen L. (2014). A lower haemoglobin level predicts a worse survival of patients with advanced gastric cancer. *Clinical Oncology*.

[44] Zhang J., Li S.-Q., Liao Z.-H. (2017). Prognostic value of a novel FPR biomarker in patients with surgical stage II and III gastric cancer. *Oncotarget*.

[45] Squires M. H., Kooby D. A., Poultsides G. A. (2015). Effect of perioperative transfusion on recurrence and survival after gastric cancer resection: a 7-institution analysis of 765 patients from the US gastric cancer collaborative. *Journal of the American College of Surgeons*.

[46] Kanda M., Kobayashi D., Tanaka C. (2016). Adverse prognostic impact of perioperative allogeneic transfusion on patients with stage II/III gastric cancer. *Gastric Cancer*.

[47] Cui J., Deng J., Ding X. (2016). Blood transfusion does not affect survival of gastric cancer patients. *Journal of Surgical Research*.

[48] Grasso M., Pacella G., Sangiuliano N., De Palma M., Puzziello A. (2019). Gastric cancer surgery: clinical outcomes and prognosis are influenced by perioperative blood transfusions. *Updates in Surgery*.

